# Postimplementation Evaluation in Assisted Living Facilities of an eHealth Medical Device Developed to Predict and Avoid Unplanned Hospitalizations: Pragmatic Trial

**DOI:** 10.2196/55460

**Published:** 2024-12-10

**Authors:** Jacques-Henri Veyron, François Deparis, Marie Noel Al Zayat, Joël Belmin, Charlotte Havreng-Théry

**Affiliations:** 1 Presage Paris France; 2 Arpavie Issy-les-Moulineaux France; 3 Laboratoire Informatique Médicale et Ingénierie des Connaissances en e-santé (UMRS 1142) Institut National de la Santé et de la Recherche Médicale Sorbonne Université Paris France; 4 Hôpital Charles Foix Assistance Publique-Hôpitaux de Paris Ivry-sur-Seine France

**Keywords:** digital technology, unplanned hospitalization, machine learning, predictive tool, assisted living facility, eHealth, pragmatic trial, artificial intelligence, AI, gerontology, older people, aging, quality of life, uncontrolled multicenter trial, France, smartphone, app, telehealth, telemonitoring, remote monitoring of patients, electronic patient-reported outcome measure, ePROM

## Abstract

**Background:**

The proportion of very old adults in the population is increasing, representing a significant challenge. Due to their vulnerability, there is a higher frequency of unplanned hospitalizations in this population, leading to adverse events. Digital tools based on artificial intelligence (AI) can help to identify early signs of vulnerability and unfavorable health events and can contribute to earlier and optimized management.

**Objective:**

This study aims to report the implementation in assisted living facilities of an innovative monitoring system (Presage Care) for predicting the short-term risk of emergency hospitalization. We describe its use and assess its performance.

**Methods:**

An uncontrolled multicenter intervention study was conducted between March and August 2022 in 7 assisted living facilities in France that house very old and vulnerable adults. The monitoring system was set up to provide alerts in cases of a high risk of emergency hospitalization. Nurse assistants (NAs) at the assisted living facilities used a smartphone app to complete a questionnaire on the functional status of the patients, comprising electronic patient-reported outcome measures (ePROMs); these were analyzed in real time by a previously designed machine learning algorithm. This remote monitoring of patients using ePROMs allowed notification of a coordinating nurse or a coordinating NA who subsequently informed the patient’s nurses or physician. The primary outcomes were the acceptability and feasibility of the monitoring system in the context and confirmation of the effectiveness and efficiency of AI in risk prevention and detection in practical, real-life scenarios. The secondary outcome was the hospitalization rate after alert-triggered interventions.

**Results:**

In this study, 118 of 194 (61%) eligible patients were included who had at least 1 follow-up visit. A total of 38 emergency hospitalizations were documented. The system generated 92 alerts for 47 of the 118 (40%) patients. Of these 92 alerts, 46 (50%) led to 46 health care interventions for 14 of the 118 (12%) patients and resulted in 4 hospitalizations. The other 46 of the 92 (50%) alerts did not trigger a health care intervention and resulted in 25 hospitalizations (*P*<.001). Almost all hospitalizations were associated with a lack of alert-triggered interventions (*P*<.001). System performance to predict hospitalization had a high specificity (96%) and negative predictive value (99.4%).

**Conclusions:**

The Presage Care system has been implemented with success in assisted living facilities. It was well accepted by coordinating nurses and performed well in predicting emergency hospitalizations. However, its use by NAs was less than expected. Overall, the system performed well in terms of performance and clinical impact in this setting. Nevertheless, further work is needed to improve the moderate use rate by NAs.

**Trial Registration:**

ClinicalTrials.gov NCT05221697; https://clinicaltrials.gov/study/NCT05221697

## Introduction

The aging population is increasing at an unprecedented rate, which is a major issue for many developed countries, particularly France [1,2]. The global population aged over 60 years is predicted to increase more than 4-fold by 2050, reaching 2 billion, an increase from its current figure of 605 million, and it will then account for 16% of the world’s total population [3]. This demographic shift represents a significant challenge for health care systems and public policies. They must adapt to address the unique requirements of this aging population, who are affected by frailty, dependency, and comorbidities more frequently than others. This calls for the involvement of a wide range of health care professionals, caregivers, and family members [4]. Moreover, the number of emergency department (ED) visits and unplanned hospitalizations in this population has lately increased significantly [5]. In France, for example, over 3 million individuals aged 70 and older are hospitalized each year [6]. Hospitalization of the older population leads to many adverse events, such as confusion, falls, drug iatrogenic reactions, nosocomial infections, and hospitalization-related dependence, resulting in loss of autonomy in daily living activities [7,8].

Although providing medical assistance to this population is currently challenging, there are initiatives to expand the range of services available to them, such as assisted living facilities. These communities provide support to older adults throughout their lives by adopting a strategy to prevent functional decline [9]. In France, more than 120,000 older adults reside in assisted living facilities [10]. However, residents in assisted living facilities have a higher risk of hospitalization and ED visits [11]. A study conducted in Canada found that hospitalization rates in assisted living facilities (36.1%) were greater than hospitalization rates in nursing homes (10.7%) [12]. Efforts have been made for several years to improve the organization of assisted living facilities to reduce the risk of hospitalization, facilitate the involvement of professionals, and coordinate care pathways of patients [13].

Prevention of unplanned hospital admissions is a major concern for the care of older adults, as a large proportion of ED visits could be avoided [14]. Digital tools based on artificial intelligence are used in the health care industry, such as motion sensors and real-time monitoring devices. These technologies can detect early signs of frailty, such as reduced mobility, sleep disorders, and alterations in eating habits. They have the potential to improve the quality of life and predict the risk of hospitalization for older adults [15]. Several systems have been developed in Europe to assist frail older adults and people with disabilities [16,17].

Efforts are currently underway to promote the development and diffusion of digital technology for identifying and preventing frailty among older adults in assisted living facilities. We developed Presage Care (PC), a remote monitoring system for patients based on electronic patient-reported outcome measures (ePROMs) with a machine learning algorithm to predict the risk of emergency hospitalizations and a prediction window of 7-14 days [18] (Multimedia Appendix 1). PC is a Conformité Européene–marked medical device. Sensitivity and specificity are 83% and 86%, respectively [19]. The alerts provided by this system enable health care professionals to act at the right time, before health conditions deteriorate significantly. When necessary, a hospitalization can be scheduled in an appropriate department. Hospitalization prediction is a real step forward, given the serious consequences of emergency hospitalization for older people and the increased risk of morbidity and mortality [6].

Few studies in the literature have investigated the importance of community-based strategies in preventing avoidable hospitalizations of older adults [20-22].

The purpose of this pragmatic study was to evaluate the feasibility of this remote monitoring system for patients using ePROMs in an assisted living facility. Additionally, we evaluated the effectiveness and impact of the system in reducing and predicting emergency hospitalizations. The aim was also to evaluate alert-triggered health interventions (ATHIs).

## Methods

### Study Design and Recruitment

This multicenter, uncontrolled pragmatic trial was conducted in 7 assisted living facilities (part of the Arpavie Group), located in France, from March 1, 2022, to August 31, 2022.

To be eligible for the PC system, participants had to be aged 65 years or older, living in one of the participating assisted living facilities, and receiving the help of a health professional. The demographic data of the participants (age and gender) were collected by the nurses and health care assistants.

### Intervention

**Figure 1:**
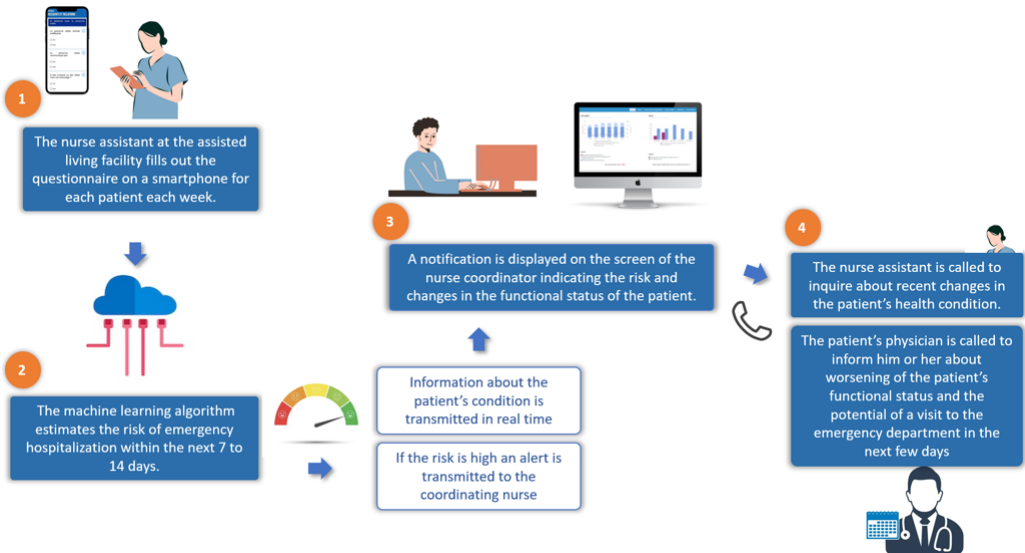
Organization of the system.

The intervention is summarized in Figure 1. The nursing assistants (NAs) at the assisted living facilities were equipped with a smartphone app. They were invited to fill out an ePROM-type questionnaire after home visits on a weekly basis via the smartphone app. The list of the 23 ePROMs recorded by the NAs is reported in Multimedia Appendix 2. Regular training was provided on the use of the app. The questionnaire focused on functional and clinical autonomy (ie, activities of daily life), possible medical symptoms (fatigue, falls, and pain), changes in behavior (recognition and aggressiveness), and communication with the NA, nurse, or family members. This questionnaire was composed of very simple and easy-to-understand questions, giving a global view of the person’s condition. For each of the 23 questions, a yes/no answer was requested. Data recorded by NAs were sent in real time to a secure server to be analyzed by our machine learning algorithm, which predicted the risk level within 14 days and displayed it on a secure web-based medical device.

Importantly, when the algorithm predicted a high risk level, an alert was displayed in the form of a notification on the screen to the coordinating nurse or the coordinating NA at the assisted living facility and to the physician. This risk notification was accompanied by information about recent changes in the patients’ functional status, identified from the NAs’ records, to assist the coordinating nurse or NA in interacting with other health professionals and family caregivers.

In case of an alert, the coordinating nurse or NA called the professional to inquire about recent changes in the patient’s health condition and to remove any doubts, and the NA could then decide to ask for a health intervention according to a health intervention model developed before the start of the study. This ATHI consisted of calling the patient’s physician and informing him or her about a worsening of the patient’s functional status and the potential risk of an ED visit in the next few days according to the eHealth system algorithm. The ATHI was performed with the available resources of the health system and not by the physicians or nurses who participated in the study (Figure 1). After its completion, a nurse reported the ATHI on a dedicated electronic reporting platform. These interventions were classified into 3 categories: social, paramedical, and health. The social category included actions such as social assistance, reassessment, reinforcement of home help services, and the provision of workshops in partnership with the beneficiary. The paramedical category included occupational therapy as well as exchanges between the home nursing service and private nurses. The health category included home visits, scheduled hospitalizations, and the geriatric mobile team. Nurses could also report if the ATHI was not relevant.

The alerts were only presented when they were trustworthy (ie, when there were enough data); however, to minimize a possible loss of opportunity, the person’s condition and risks (geriatric related and health related) were passed on to the coordinating nurse, who could then assess the gravity of the person’s situation.

### Outcomes

#### Feasibility and Organizational Aspects

The primary outcomes were related to use of the system and are described in Textbox 1 [22,23].

The outcome targets were defined according to a previous study that highlighted the acceptability of the device to health care professionals and its effectiveness in real-life use by older adults living at home (Textbox 2) [19].

**Primary outcomes.**

**Proportion of eligible professionals who used the system**

Eligible professionals were defined as the total number of trained people at the home nursing service; a rate of use of more than 70% was considered to be acceptable.

**Median completion time**

A completion time under 2 minutes was considered to be acceptable.

**Proportion of alerts that led to an alert-triggered health intervention**

If 70% of alerts led to an intervention, it was considered to be acceptable.

**Outcome targets.**

**Regularity (proportion of professionals with regular use according to the standards)**

- A result of 100% for nurse assistants meant that they all collected questionnaire data over 4 consecutive weeks, while 0% meant that none collected data over 4 consecutive weeks.
- A result of 100% for coordinating nurses meant that they used the Presage Care web application at least 3 times a week over 4 consecutive weeks.

**Performance (effectiveness and efficiency of artificial intelligence in risk prevention and detection in practical, real-life scenarios)**

- We monitored the eHealth system alerts’ ability to predict emergency hospitalizations by assessing the true negative rate (ie, specificity) and false negative rate. The true positive rate (ie, sensitivity) was also monitored.

**Delay between the alert and the intervention**

- A delay of less than 4 days was considered to be acceptable [24].

#### Clinical Effect

The secondary outcome was the rate of emergency hospitalizations after an ATHI compared to the rate of emergency hospitalizations after no ATHI .

### Statistical Analysis

Quantitative data were described using the mean and SD, or the median and IQR in cases of nonnormal distribution. Categorical data were presented as frequencies and percentages. A 2-tailed Student *t* test or Wilcoxon nonparametric test were used to compare continuous variables, whereas the *χ*^2^ test or Fisher exact test were used for categorical variables. For each alert (by the index test), negative likelihood ratios were estimated relative to an emergency hospitalization, which was considered the target condition of the reference standard. *P* values <.05 were considered statistically significant. Statistical analyses were performed using Stata (version 16; StataCorp LLC).

### Ethical Considerations

The research protocol was approved by the national French ethics committee for biomedical research, the Comité de Protection des Personnes, and the French Agency for the Safety of Health Products (2021-A02131-40–CPP 1-21-072/21.02093.000019). The protocol was presented to professional and patient representatives from the Arpavie Group (the Conseil des Résidents et des Familles). Participants, the health care professionals, and the assisted living facilities’ managers were informed about the nature and purpose of the study and provided their written consent accordingly. Data storage was certified according to the ISO/IEC 27001 standard. The data were not accessible to the NAs, and the nurse coordinator and physcians could access the data only after validation with a one-time password. The patients were permitted to benefit from the service during the study; however, they received no additional compensation.

## Results

### The Inclusion of Centers and Patients

Among the 194 older adults in assisted living facilities who were eligible for medical device follow-up, 118 (118/194, 60.8%) were enrolled in the study and followed up at least once. The proportion of participants included in the study from each inclusion site varied by site; for example, participants from one of the sites represented 10.3% (20/194) of the total, while those from another site represented 44.8% (87/194); membership thus differed according to the facility, showing that it was based on the free choice of the older adults.

### Characteristics of the Health Professionals and Participants

The mean age of the older adults was 82.55 (SD 9.19) years, with 84 of 118 (71.2%) of them being women. Out of a total of 33 eligible professional caregivers, 27 were trained (82%) and filled out questionnaires at least 1 time. Furthermore, all (20/20, 100%) coordinating nurses were trained.

### Feasibility of the Implementation of the Device in an Assisted Living Facility

#### Completion Among Professionals and Nursing Coordinators

Among the 27 health care professionals, 7 were (26%) active during the study and used the system regularly. Regularity of use was 100% in this group. Among the 20 coordinating nurses, 9 (45%) performed regular analyses. All 9 of these coordinating nurses used the system with 100% regularity. All the facilities had some health care professionals who did not participate, and several of them had in fact left their facility during the study for another job.

#### Completion Time

A total of 1789 follow-ups were monitored, with a median duration for completion of 102 (IQR 86) seconds. The minimum time was 45 seconds and the maximum was 432 seconds (Table 1).

**Table 1:** Duration of follow-ups: about 80% of follow-ups were completed in less than 150 seconds.

Duration, s	Follow-ups (n=1789), n (%)
45-79	513 (28.7)
80-114	547 (30.6)
115-149	322 (18)
150-184	107 (6)
185-219	84 (4.7)
220-254	80 (8.5)
255-298	90 (5)
290-324	9 (0.5)
325-359	18 (1)
360-394	17 (1)
395-429	1 (0.1)
430-464	1 (0.1)

#### Performance of the eHealth System

During the study, a total of 92 alerts were generated by the medical device, corresponding to 5.1% (92/1789) of the follow-ups. Out of 38 emergency hospitalizations, 9 (24%) did not have any alerts in the preceding 14 days, whereas 29 (76%) were preceded by alerts within that timeframe (*P*<.001; Table 2).

The sensitivity and specificity of the alerts to predict emergency hospitalizations occurring within 14 days after the alerts were 76% and 96%, respectively. The positive and negative likelihood ratios were 17.2 and 0.36, respectively. The true positive rate was 30% and the true negative rate was 99.4%.

**Table 2:** Performance of the predictive system within 14 days after a regular follow-up. There were 92 alerts generated for 1789 follow-ups (5.1%).

Characteristics			Alert		No alert	*P* value	
**Emergency hospitalizations within 14 days after follow-up, n (%)**							<.001
	Yes (n=38)	29 (76.3)		9 (24.7)			
	No (n=1751)	63 (3.6)		1688 (96.4)			

### Health Interventions After an Alert Display

The system generated 92 alerts for 47 (47/118, 39.8%) patients. Out of these alerts, 46 (46/92, 50%) resulted in health care interventions for 14 (14/118, 11.9%) patients. The social category represented 41% (19/46) of the interventions, the paramedical category represented 24% (11/46), and the health category represented 35% (16/46). The average response time for an alert was 2 days. There was 1 alert (1/92, 1%) that was evaluated as not being an alert after a call to the nurse.

### Clinical Effect of ATHIs: Health Care Intervention and Hospitalization

**Figure 2:**
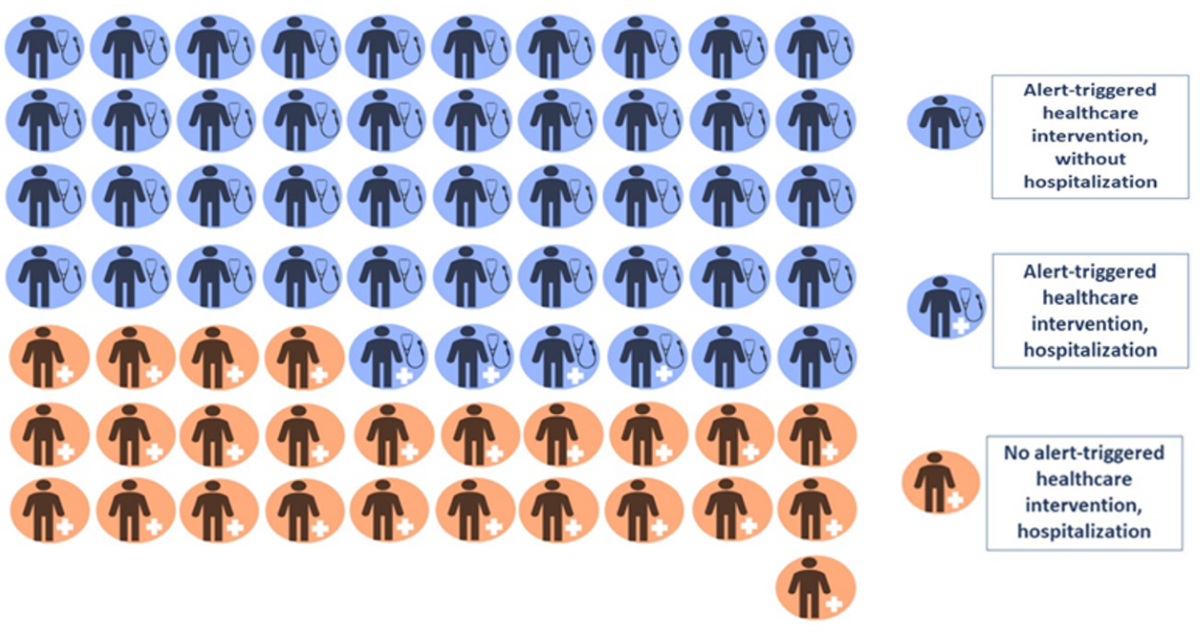
Alert-triggered interventions and hospitalizations after alerts. We monitored patient pathways after each of the 92 alerts: of the 29 hospitalizations (marked by crosses), 25 (86%) did not receive any intervention support from nurses (labeled in orange). For 42 of 46 (91%) alerts that were followed by an intervention, there was no subsequent hospitalization (labeled in blue).

The total of 46 ATHIs resulted in 4 (4/46, 9%) hospitalizations, and the other 46 alerts, which did not trigger health care interventions, resulted in 25 (25/46, 54%) hospitalizations (*P*<.001) (Table 3, Figure 2). The patient’s nurse or general practitioner carried out these health care interventions. Of the 47 patients who received alerts, 21 were hospitalized, 19 of whom (90%) did not receive any ATHI. When there was no ATHI, 58% of patients (19/33) were hospitalized compared to 14% of patients when there was an ATHI (2/14; *P*<.001).

**Table 3:** Hospitalizations of patients that occurred within 14 days of alerts generated by the eHealth system, according to whether there was an alert-generated health intervention (ATHI) among the 47 patients for which Presage Care system provided alerts. The results are expressed as number of events and number of patients, since many patients experienced several alerts and several hospitalizations during the study.

			No ATHI, n (%)		ATHI, n (%)		*P* value
**Events**							<.001
	Alerts (n=92)	46 (50)		46 (50)			
	Emergency hospitalizations (n=29)	25 (86)		4 (14)			
**Patients**							<.001
	Patients with alerts (n=47)	33 (70)		14 (30)			
	Patients with emergency hospitalization (n=21)	19 (90)		2 (10)			

## Discussion

### Principal Results

The aim of this study was to evaluate the feasibility of the PC system in reducing emergency hospitalizations among older adults in assisted living facilities. The study showed that the system was accepted and used by a part of the independent-living professionals, with a use rate of 26% (7/27) for NAs and 45% (9/20) for coordinating nurses. For those who were using the system, regularity of use was excellent, with a rate of 100% for NAs and coordinating nurses. The median completion time to fill out the questionnaire was 102 seconds, which is acceptable. The results showed that 5% (89/1789) of follow-up visits had a completion time of 5 minutes or more; this much longer time was because of a professional having a discussion with a participant during the process of collecting an observation. System performance was very good, as specificity was 96% and the true negative rate was 99.4%.

The rate of interventions after an alert was moderate (46/92, 50%), and in half of the cases, an intervention was absent; we need to understand the reasons for this. However, the implementation of interventions after receiving alerts resulted in the successful prevention of hospitalization in over 91% of instances. Hospitalizations were strongly correlated with the absence of health care interventions (25/29, 86% of hospitalizations; *P*<.001). Moreover, the diagnostic capability of PC in assisted living facilities was high. When no intervention was made after an alert, 58% of the older adults were hospitalized.

These results suggest that greater completion rates by coordinating nurses would have a major clinical impact, helping to avoid as many emergency hospitalizations as possible.

The PC medical device successfully bridges the access gap associated with this type of device. The predictive performance of the device is consistent with prior published real-life studies on this device [19].

### Comparison With Prior Studies

The times for filling out the questionnaire and the completion rate are consistent with a previous study on this system; however, they surpass the outcomes in other studies that highlighted issues such as long completion times and poor usability of ePROMs for caregivers and clinicians [25].

Early identification of symptoms enables “more effective monitoring and more appropriate management, leading to very concrete and sometimes significant clinical impacts, such as prolonging patients’ lives and improving their quality of life” [26]. This effect was described in particular by a meta-analysis of 28 clinical trials, which found an overall improvement in the survival of older participants who participated in a gerontological assessment with long-term follow-up by specialized teams [27]. This meta-analysis, published in the *Lancet*, showed that, depending on the type of program proposed, these interventions could reduce mortality by 14% and hospitalization rates by 12%. There was also a 26% increase in home survival, a 41% improvement in cognitive function, and a 72% improvement in functional status [28].

Compared with the literature, our medical device seems to have achieved particularly good results in preventing emergency hospitalization in older adults.

### Limitations of the Study

The study encountered several limitations: it was conducted during a short period and was not a randomized controlled trial with control and intervention groups. Moreover, no analysis was carried out to assess the impact of the type of professional (nurse, home aide) on the rate of completion of the questionnaire and the accuracy of the information. The relatively low participation rate among NAs was a major limitation of this study, which may be due in part to high staff turnover, and it is likely that people with short-term plans to leave the facility had little motivation to participate. In future studies, this point should be taken into account and could be ameliorated by renewed teaching for newly arrived staff or by offering incentives, rewards, or reminders. In addition, this study does not precisely describe the impact of the delay between the alert and the health intervention. Finally, the study was not controlled, and a large-scale randomized control trial is needed to measure the added value of the system in preventing emergency hospitalizations among frail older adults.

### Perspectives

A randomized controlled study should be done to demonstrate how health care professionals use the new information provided by this system and examine its clinical efficacy and cost-effectiveness. The safety profile of the medical device could be assessed with a comparative analysis of adverse events or complications observed prior to and following its implementation, with the aim of identifying potential risks and side effects associated with the device. Further analysis is needed to assess the project’s sustainability in a context where professional turnover is high.

### Conclusion

This study demonstrates the efficacy of the PC system in minimizing unplanned hospitalizations of older adults in assisted living and assessed the predictability of the alerts generated by the system. Improvements in the regularity of use of the system by health care professionals should be feasible in future research. These encouraging results promise to be extendable to a larger population.

## Appendix

Multimedia Appendix 1 Presage Care, a remote monitoring system for patients using electronic patient-reported outcome measures with a machine learning algorithm to predict the risk of emergency hospitalizations with a prediction window of 7-14 days.

Multimedia Appendix 2 List of electronic patient-reported outcome measure items recorded and their completeness.

## Abbreviations

ATHI: alert-triggered health intervention
ED: emergency department
ePROM: electronic patient-reported outcome measure
FNR: false negative rate
NA: nursing assistant
PC: Presage Care
TPR: true negative rate

## Data Availability

The data that support the findings of this study are available from the corresponding author, JHV, upon reasonable request. Restrictions apply to the availability of the data collected by the nursing assistants and to the algorithm that was used in this study, which has been patented.
