# Supplementary material for: Postimplementation Evaluation in Assisted Living Facilities of an eHealth Medical Device Developed to Predict and Avoid Unplanned Hospitalizations: Pragmatic Trial
Source: J Med Internet Res. 2024 Dec 10;26:e55460. doi: 10.2196/55460 (PMC11668978; doi:10.2196/55460)
Supplement: Multimedia Appendix 1 [file jmir_v26i1e55460_app1.docx]

This figure is a simplified diagram of a data processing system for the determination of a risk factor of an imminent transfer of an individual 110 to emergency hospitalization. The data processing system comprises a computer server (120) provided with a microprocessor and with a computer memory in which is stored an automatic learning computer algorithm allowing determining a value representative of the risk of the individual (110) having emergency hospitalization in the near future, corresponding in general to the next 14 days. Later on, this value is called risk factor.

Each status sheet can be filled by an home aide (115) vising the individual (110). The computer server (120) is connected to a database (122) storing the status sheets established beforehand for a group of persons and a database (124) storing the dates of transfers of this group of persons to emergency hospitalizations. From the status sheets and the dates of transfers of the group of persons to the emergency hospitalizations, parameters of the automatic learning computer algorithm are generated by means (126) for generating said parameters. To this end, the computer server (120) may be configured to generate said parameters. To predict the risk of transfer to emergency hospitalizations, the data processing system comprises means (128) for determining the risk factor through the analysis of a plurality of status sheets of the individual (110) thanks to the algorithm whose parameters have been generated beforehand.

As soon as the value of the risk factor exceeds a predetermined threshold, an alert is generated by means for generating (130) an alert of the data processing system (100). In particular, this alert may be sent to an intervention platform (140) (coordinating nurses) in order to be able to rapidly take charge of the individual (110). For the regular filling of the status sheet, the system (100) comprises a device (150) for filling a status sheet which is generally a smartphone or a tablet used by the home aides (115). In order to collect the data originating from the filling device (150) to transmit them to the computer server (120), the system (100) also comprises a collection terminal comprising wireless communication means for receiving these data.

Once status sheets (questionnaire) have been recorded for the individual (110), the analysis of the evolution of the monitoring indicators can be performed by the automatic computer algorithm whose parameters have been generated beforehand. In order to improve the prediction of emergency hospitalization in the near future, the data processing system (100) also comprises a database (180) storing the geolocated and dated epidemiological information relating to the temperature of the commune, relating to influenza-like and/or acute diarrhea illnesses. Furthermore, as soon as the individual (110) has been hospitalized, the date of transfer of the individual (110) to the emergency hospitalization is recorded in the database (124).
